# The truth about modelling – disordered eating, body image, abuse and more: A content analysis among professional fashion models

**DOI:** 10.1192/j.eurpsy.2024.486

**Published:** 2024-08-27

**Authors:** N. Bogár, F. Túry, P. Kővágó

**Affiliations:** ^1^Institute of Behavioural Sciences, Semmelweis University; ^2^Institute of Psychology, Pázmány Péter Catholic University, Budapest, Hungary

## Abstract

**Introduction:**

The escalating demand for models to uphold a slim physique and extremely small measurements could play a pivotal role in contributing to the onset of eating disorders, in their clinical or subclinical forms.

**Objectives:**

The study aimed to explore models’ relationship with food, exercise, body image, industry members, experience of abuse and other related factors through self-narrated reports. To our knowledge, this study involved a larger number of multicultural female models than any previous qualitative research and is the first-ever study to use content analysis for the assessment of ED-like symptoms and body image disturbances in this population.

**Methods:**

87 models’ data was analyzed. Snowball sampling was used. Semi-structured interviews targeted models’ careers, attitudes towards the fashion industry, their body image, eating, exercising and dieting habits, etc. Thematic content analysis was performed on the transcripts of the interviews. A coding booklet was developed containing instructions on 31 codes. The codes developed for the analysis included calorie restriction, weight gain, loss of control, laxative abuse, self-induced vomiting etc. They also included specific symptoms of eating and/or body image disorders. the analyses were conducted using relative frequencies. The absolute frequency of the codes was divided by the wordcount corresponding to the interview.

**Results:**

The mean BMI of the subjects was 16.8 (SD= 1.30, range 13.58- 19.37). 44.7% of the models reported BMI of between 18.5 and 17.0, and 21.2% were under 17.0. Body image disorder symptoms were expressed by 63.10% of the models, and 36.90% have referred to eating disorders. The most referenced code was statements about the subjects’ bodies (95.24% neutral, 89.29% negative, 64.29% positive statements). Statements about eating included 96.43% neutral and 45.24% negative claims. Monotrophic eating occurred in 27.38% of the answers, and 40.48% claimed to have used extreme calorie restriction. Juice fasting was occurrent amongst 3.57% of the interviewees. 22.62% have lost control over their food intake. 83.33% of the participants received criticizing comments on their bodies and such individuals talk negatively significantly more often about eating. Those individuals who engage in psychotherapy (16.67%) show significantly fewer signs of body image disorders, however, talk significantly more about eating disorder-related content.

**Conclusions:**

The persistent expectation for thinness in the fashion industry elevates the likelihood of eating disorders and body image disorder development among models. The current study aims to offer insights into prevention strategies.

**Disclosure of Interest:**

None Declared

